# New prediction equations for knee isokinetic strength in young and middle-aged non-athletes

**DOI:** 10.1186/s12889-023-17478-7

**Published:** 2023-12-21

**Authors:** Ye Zhang, Kang Chen, Kun Liu, Qingliang Wang, Yuhui Ma, Bo Pang, Lihua Huang, Yanhong Ma

**Affiliations:** Department of Rehabilitation Medicine, Shanghai Sixth People’s Hospital Affiliated to Shanghai Jiao Tong University School of Medicine, Shanghai, 200233 China

**Keywords:** Prediction equations, Muscle weakness, Isokinetic, Peak torque, Knee strength

## Abstract

**Background:**

This study aimed to develop alternative prediction equations to predict isokinetic muscle strength at 60°/s based on anthropometric characteristics, including body mass, height, age, and sex for young and middle-aged non-athlete populations.

**Methods:**

Three hundred and thirty-two healthy non-athletic participants (174 females, 158 males) between 20 and 59 years underwent a 60°/s isokinetic knee joint concentric contraction test. Forty people were randomly selected for retesting to assess the reliability of the isokinetic instrument. Multivariate linear regression was used to establish extension peak torque (EPT) and flexion peak torque (FPT) prediction equations. Sixty extra participants were used individually to validate the prediction equations, and Bland Altman plots were constructed to assess the agreement of predicted values with actual measurements.

**Results:**

The result demonstrated that the instrument we used has excellent reliability. The multivariable linear regression model showed that body mass, age, and sex were significant predictors of PT (EPT: Adjusted R^2^ = 0.804, *p* < 0.001; FPT: Adjusted R^2^ = 0.705, *p* < 0.001). Furthermore, the equations we established had higher prediction accuracy than those of Gross et al. and Harbo et al.

**Conclusion:**

The equations developed in this study provided relatively low bias, thus providing a more suitable reference value for the knee isokinetic strength of young and middle-aged non-athletes.

## Background

Muscle strength is the main component of muscle-skeletal exercise and is core to physical performance and motor skill development [1]. Muscle atrophy is a common dysfunction of people suffering from long-term health issues, such as complex trauma requiring long-term immobilization, long-covid, cancer, and sarcopenia [2–5]. This dysfunction increases the risk of weakness, fracture, hospitalization, and death, causing an economic burden on individuals, families, and society, especially for young and middle-aged people who need to take on more social responsibilities.

Knowledge of patients' muscle strength will help clinicians set reasonable rehabilitation goals for patients and evaluate their recovery progress. Bilateral comparison is a commonly used method in clinical practice to evaluate the degree of muscle strength decline [6]. For unilateral knee joint injuries, one of the main goals of rehabilitation is to establish a strength level similar to that of the uninjured side. Some studies recommend that the affected side's muscle strength should reach at least 80% to 90% of that of the healthy side before returning to the sports field [7–9]. However, the premise for applying this method is that the target side for comparison has not experienced muscle atrophy. In reality, for patients with long-term health-related issues, both limbs often experience varying degrees of muscle atrophy [10], so the rehabilitation goals established through bilateral comparison are not suitable for them. Knowing patients' muscle strength before injury will also help clinicians set reasonable training goals for patients. Unfortunately, due to the high cost and lack of portability of professional muscle strength testing equipment such as isokinetic dynamometry [11], the general population rarely tests muscle strength when in a healthy state, so obtaining muscle strength data before atrophy in patients is often challenging. In this context, if there is a normative reference value or prediction model of knee strength level, it will provide great convenience for clinicians to set reasonable training goals for patients.

Isokinetic dynamometry is one of the popular tools for evaluating muscle strength in clinical and research settings [12–14], and 60°/s is the commonly used angular velocity to test the maximal strength of the knee joint. Although many researchers have reported reference data for isokinetic strength in some specific populations [15–19]. It is difficult to conclude generally applicable reference values from these data because of strict application condition limitations for specific reference values such as specific age range, height, body mass, sex, and sports participation. In contrast, exploring the correlation between anthropometric characteristics and muscle strength in healthy participants by multivariable linear regression and building prediction equations for muscle strength may apply to a broader clinical population.

Indeed, several studies have clarified the value of anthropometric variables such as height, body mass, age, and body fat percentage to predict strength and have provided several predictive equations for normal isokinetic strength of the knee [20–22]. However, during our application of their equations, we found that the actual measured values of knee isokinetic strength differed significantly from the predicted values. Among these equations, Gross et al.'s and Harbo et al.'s equations provided the prediction equations of knee flexion and extension strength, and both used four commonly used anthropometric indicators as independent variables, namely age, sex, body mass, and height, which was consistent with our study [20, 22]. Considering these, the objective of this study was to conduct multiple linear regression between the peak torque (PT) of the isokinetic knee flexor and extensor measured at 60°/s angular velocity and commonly used anthropometric data such as height, body mass, sex, and age, develop new alternative prediction equations for knee strength of young and middle-aged non-athletes, and compare the new prediction equations with the existing ones of Gross et al. and Harbo et al.

## Methods

### Participants 

A priori power analysis indicated that a sample of 49 patients would provide 90% statistical power, with α set at 0.05, to detect a medium effect size (f^2^ = 0.35) for a regression with four predictors. To improve the stability of the equations, we have expanded the sample size. The study sample for establishing the prediction equation was 332 healthy young and middle-aged non-athletes (174 females and 158 males) aged between 20 and 59 years. The average age of female participants was 41.39 ± 10.77 years, and the average age of male participants was 40.19 ± 10.38 years. After the prediction equations were established, sixty healthy participants were included for validation of the prediction equations. The validation sample met criteria similar to those for building predictive equations. The exclusion criteria are any neurological, endocrine, mental, cardiorespiratory disease, lower-extremity injury, or history of drug or alcohol abuse. All participants were asked to refrain from vigorous physical activity for 48 h before the assessment. The study was approved by the Ethics Committee.

### The measure of anthropometric characteristics

Before the muscle strength test, each participant reported their age information and measured their height and body mass with a medical height and body mass measuring instrument (SH-200G, Shanghe Electronic Technology Co., Ltd., Zhengzhou, China). When measuring height and body mass, participants were asked to remove shoes, socks, and excess clothing and only wear shorts and a vest. The participants have their two heels pressed against each other, their hands relaxed on both sides of the body, and their eyes looking forward horizontally. In addition, participants were asked to hold their breath after taking a deep breath and measure the distance from the highest point of their head to the bottom of their heels. Participants can only leave after the testers have recorded their height and body mass values [23]. The statistical data of anthropometric characteristics of 332 participants are grouped by age and sex, as shown in Table 1. The sample characteristics of the validation equation are in Table 2.

**Table 1:** Subgroup analysis for pooled sensitivity of CL Detect rapid test for CL

**Sex**	**Decade of Age (yr)**	**n**	**Age (yr)**	**Body Mass (kg)**	**Height (cm)**
Female	20-29	46	25.55±1.85	55.96±8.10	165.22±6.46
	30-39	44	33.43±2.90	56.36±9.05	163.43±6.66
	40-49	44	43.05±2.82	60.27±6.62	163.25±4.28
	50-59	40	55.45±2.63	59.13±8.68	160.58±6.68
	20-59	174	41.39±10.77	57.88±8.29	163.20±6.26
Male	20-29	49	25.21±2.84	73.57±11.33	177.18±7.07
	30-39	40	34.22±3.00	75.28±10.55	176.38±6.98
	40-49	39	43.09±2.49	77.69±10.87	173.77±4.99
	50-59	30	54.07±2.80	73.73±10.24	171.47±4.38
	20-59	158	40.19±10.38	75.05±10.85	175.05±6.46

Data are expressed as mean and standard deviation

**Table 2:** Anthropometric characteristics data of the validation subjects (*n*=60)

Decade of Age (yr)	n	Age (yr)	Body Mass (kg)	Height (cm)
20-29	20	26.90±1.83	65.30±14.24	170.50±10.74
30-39	23	32.17±2.04	66.52±14.18	171.22±9.77
40-49	9	43.11±3.30	70.89±18.48	168.89±9.52
50-59	8	53.25±3.06	70.13±10.25	171.63±8.00
20-59	60	34.87±9.18	67.25±14.15	170.68±9.58

Data are expressed as mean and standard deviation

### Knee strength testing

The isokinetic dynamometer NX A8-3 (Yikang, Guangzhou, China) was used to test knee joint strength of random side (determined by drawing lots) in all participants. Three days before formal testing, participants visited the testing sites to familiarize themselves with the testing procedure and to experience the contraction pattern. Before the isokinetic test, the participants were informed of the test process in detail again and warmed up with a light load (25 watts, 50 to 60 RPM) for 5 min on a cycle ergometer (Nuocheng, Shanghai, China). During the test, participants were seated in the dynamometer with hips flexed to 90° and the lateral femoral condyles aligned with the axis of the power head, and the participant's trunk, pelvis, test side thigh, and non-test side calf were well fixed [24]. The knee's range of motion was set to 10° extension to 90° flexion, and gravity correction was performed to correct the effects of calf and power arm weight on knee flexion peak torque (FPT) and extension peak torque (EPT). Participants underwent formal testing at an angular velocity of 60°/s (5 repetitions). The value recorded was the individual muscle contractions eliciting the highest PT throughout the test, expressed in absolute values (Nm). Before the formal test, four adaptation exercises (consisting of three submaximal and one maximal isokinetic contractions) were carried out at the same angular velocity. During each test, the researchers gave consistent verbal commands, such as "as hard and fast as possible," to ensure participants provided maximum effort during the test. Forty participants were selected from the research group through simple random sampling to undergo two identical strength tests, one week apart, with the same procedure. The same researcher conducted the tests to optimize the accuracy of the measurements. The rest of the participants had only one strength test.

### Statistical analysis

In this study, PASS15.0 software was used to calculate the sample size. SPSS 20.0 software (IBM Corporation, NY, USA) was used for statistical processing and analysis of test data. Measurement data were expressed by mean ± standard deviation (SD). A two-way random model (type: absolute agreement) of the intraclass correlation coefficients (ICC) and the 95% confidence interval (CI) of the ICC were used to analyze the test–retest reliability between the two measurements, and the values of the ICC were interpreted according to the criteria in the recent guideline [25, 26]. Bland Altman plots were used to assess the level of agreement between measured PT values and those predicted by the regression equations. When conducting Bland Altman analysis, SPSS 20.0 software was used to draw residual plots to test the homoscedasticity of residuals and histograms to test the normality of residuals. The difference was statistically significant with *p* < 0.05.

The dependent variables in this study were FPT and EPT values after gravity correction at 60° / s, and the independent variables were sex (coded as a dichotomous variable, female = 0, male = 1), age, height, and body mass. Predictive equations for PT were determined using stepwise multivariable linear regression, and only statistically significant independent variables were retained in the final models. Data were checked for the satisfaction of the conditions of the regression assumptions [27]: (1) residual independence, (2) no multicollinearity problem between independent variables, and (3) residuals fit a normal distribution. In addition, the PT values measured by the participants were also validated against the equations reported by Gross et al. [20] and Harbo et al. [22]. The closer the adjusted R^2^ value of the prediction equation is to 1, the higher the independent variable's explanatory power on the dependent variable's variation.

## Results

Table 3 presents the ICC and 95% CI for ICC. The PT of knee extension and flexion at 60° / s showed excellent reliability, according to the mean value of ICC and 95% CI of ICC (> 0.90). All ICC values were significant (*p* < 0.001). The results showed that the instrument we used was reliable.

**Table 3:** Repeatable measurement of isokinetic knee flexion and extension

	**ICC**	**95% CI for ICC**	***p***
Knee extension at 60°/s	0.961	(0.927,0.979)	<0.001
Knee flexion at 60°/s	0.948	(0.905,0.972)	<0.001

*ICC* intraclass correlation coefficient, *CI* confidence interval

After testing, the data fit the regression equation assumptions, and the Durbin-Watson test values of the prediction models for EPT and FPT were 1.743 and 1.798, respectively, indicating that each observation sample was independent of the other. The residual histograms and P-P plots suggested that the standardized residuals follow a normal distribution. The maximum variance inflation factor among the predictors was 1.826, which can be considered the absence of multicollinearity among independent variables. Table 4 presents the results of the stepwise multivariable linear regression for EPT value. The regression model of EPT showed that body mass was the most significant predictor of EPT (β = 0.592, *p* < 0.001). In contrast, age (β =− 0.404, *p* < 0.001) and sex (β = 0.279, *p* < 0.001) improved the predictive ability of the equation, while height was not a significant predictor of EPT (*p* > 0.05). The prediction equation (Eq 1) for the EPT value was:

1 $${\text{EPT}}=63.085+2.057 \times \mathrm{ Body\;mass }\left({\text{kg}}\right)-1.55 \times \mathrm{Age\;}({\text{year}})+24.935\times {\text{Sex}}$$

where sex = 0 for females and 1 for males. All these predictors explained 88.40% of the variation in the PT values of knee extension (Adjusted R^2^ = 0.804, F = 453.71, *p* < 0.001).

**Table 4:** Regression analysis of the relationship between EPT and anthropometric characteristics

**Predictors**	**Unstandardized B Coefficient**	**Std. Err**	**Standardized β Coefficient**	***p***
Body Mass	2.057	0.114	0.592	<0.001
Age	-1.55	0.094	-0.404	<0.001
Sex	24.935	2.936	0.279	<0.001
Constant	63.085	7.346		

Adjusted R^2^ = 0.804; Durbin-Watson = 1.743; Maximum VIF = 1.826; *EPT* Extension peak torque, *VIF* Variance inflation factor

The regression model of FPT value was similar to the extension model (Table 5), with body mass (β = 0.509, *p* < 0.001) also being the most significant predictor of FPT. In addition, age (β =—0.364, *p* < 0.001) and sex (β = 0.319, *p* < 0.001) similarly played a role in improving the equation prediction ability, while height was also not a significant predictor of FPT like EPT (*p* > 0.05). The predictive equation (Eq. 2) for the PT value of knee flexion was:

2 $${\text{FPT}}=22.324+0.913 \times \mathrm{ Body\;mass }\left({\text{kg}}\right)-0.522 \times \mathrm{ Age\;}\left({\text{year}}\right)+22.412 \times \mathrm{ Sex}$$

where sex = 0 for females and 1 for males. 70.50% of the dependent variable knee flexion PT variation can be explained by the changes in the above independent variables (Adjusted R^2^ = 0.705, F = 265.19, *p* < 0.001).

**Table 5:** Regression analysis of the relationship between FPT and anthropometric characteristics

**Predictors**	**Unstandardized B Coefficient**	**Std. Err**	**Standardized β Coefficient**	***p***
Body Mass	0.989	0.078	0.509	<0.001
Age	-0.781	0.064	-0.364	<0.001
Sex	15.945	2.013	0.319	<0.001
Constant	29.011	5.038		

Adjusted R^2^ = 0.705; Durbin-Watson = 1.798; Maximum VIF = 1.826; *FPT* Flexion peak torque, *VIF* Variance inflation factor

**Fig. 1:**
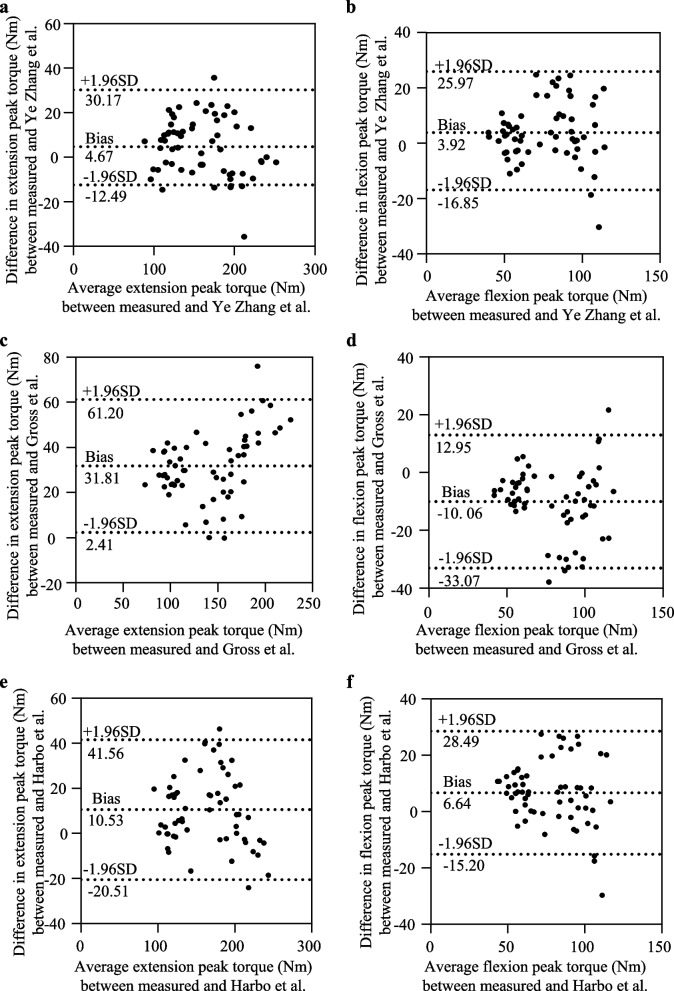
Bland Altman plots comparing measured and predicted PT using the Ye Zhang et al. (**a** and **b**), Gross et al. (**c** and **d**) and Harbo et al. (**e** and **f**) prediction equations (*n* = 60)

The Bland Altman plots (Fig. 1) compared the consistency between the predicted PT values of predicted equations and the actual measured PT values. Gross et al.'s equation underestimated the PT value of knee extension (EPT: Mean = 31.81 Nm, 95% limits of agreement (LoA): 2.41 to 61.20 Nm) and overestimated the PT value of the knee flexion (FPT: Mean = -10.06 Nm, 95% LoA:—33.07 to 12.95 Nm) (Fig. 1 c-d). Harbo et al.'s equations underestimated the PT values of knee flexion and extension (EPT: Mean = 10.53 Nm, 95% limits of agreement (LoA): -20.51 to 41.56 Nm; FPT: Mean = 6.64 Nm, 95% LoA: -15.20 to 28.49 Nm) (Fig. 1 e–f). Whereas the predicted values of our established equations showed better agreement with the prediction equations we developed (EPT: Mean = 4.67 Nm, 95% LoA:—12.49 to 30.17 Nm; FPT: Mean = 3.92 Nm, 95% LoA:—16.85 to 25.97 Nm) (Fig. a-b).

## Discussion

Accurate assessment of the degree of muscle strength reduction is of great value for recovery of function. However, there are differences between the currently available equations for reference values of knee isokinetic strength and the actual measurements. The significant contribution of this research was to establish two new alternative isokinetic strength regression models of knee joints for young and middle-aged non-athletes. Therefore, the equations established in this study can be used to predict the isokinetic strength performance of patients before injury. In addition, the results of this study also showed that the values provided by our proposed equations were closer to the measured PT values of knee extensors and flexors, presenting a lower mean error relative to the current widely applied equations provided by Gross et al. [20]and Harbo et al. [22]. Overall, this study provided alternative prediction equations for knee strength, potentially providing a more appropriate reference standard for knee isokinetic PT values, which would be beneficial for the early identification of reduced muscle strength clinically as well as for setting appropriate targets for muscle strength training.

Gross et al. established predictive equations for knee strength as early as the 1980s by regressing the PT values of knee isokinetic extension and flexion in 134 healthy participants aged 20–80 [20]. Harbo et al. recruited 178 healthy non-athletic participants aged 15–83 to establish similar predictive equations [22]. Height, body mass, age, and sex were all independent variables in their equations, which were widely used after they were established. However, we found that when applying them, there is a significant mean deviation between the predicted PT value and the actual measured value. In particular, the knee extension mean deviation reached a startling 33.81 Nm when we used Gross et al.'s equation, indicating that the equation significantly underestimated knee extension strength. The predicted values of the FPT and EPT prediction equations we established were close to the actual measurements, and the average deviations were all closer to 0 Nm, suggesting that the equations developed in this study provide more appropriate reference values for isokinetic knee strength. These differences may be due to our larger sample size and smaller age range coverage. In addition, in recent decades, the per capita intake of animal-derived foods such as meat, eggs, and milk has significantly increased [28], and the increase in dietary protein intake will also significantly improve people's thigh muscle mass and strength [29, 30]

The results of the present study should be considered in terms of its potential practical applications in evaluation. The variables in the equation, namely body mass, age, and sex, are very easy to obtain and do not rely on complex calculations or precise instruments. People with higher body mass tend to have greater knee strength [31], which is also why many studies choose the ratio of PT to body mass rather than PT value when analyzing the isokinetic knee strength characteristics of a particular class of people [32, 33]. In addition, age was a negatively predictive variable, consistent with previous studies [20, 22, 34], which may be related to the phenomenon of decreased thigh muscle mass and strength with age [35–37]. In addition, the predicted PT values of male knee flexion and extension muscles are higher than those of females, possibly due to the relatively higher proportion and mass of male muscle tissue compared to females [38–41]

Interestingly, in previous studies [20–22], height was also one of the predictive factors for knee isokinetic PT values. However, in this study, height was not a predictive factor for knee extension or flexion PT values. It is generally believed that the reason for using height as a predictor is because height, to some extent, affects the length of the tibia [42], which in turn affects the length of the arm. According to the formula "torque = force arm × force," can deduce that height may have a positive impact on PT values. The possible reason for this phenomenon is that although the higher the height, the longer the tibial length, the tibial length does not scale equidistantly with the height. Many studies have shown that there are different relationships between tibial length and different stature (short, medium, high) [43, 44]. Compared with the height, which indirectly reflects the arm of force and has certain variability, it is more suitable to use the tibial or calf length as a predictor directly.

In summary, establishing predictive equations to estimate PT accurately is crucial for identifying early knee joint strength loss and timely treatment interventions. Therefore, in future research, it is necessary to explore more suitable indicators as predictive variables, such as leg or tibia length, to improve the accuracy of the knee joint isokinetic force prediction equation. In addition, more accurate prediction equations need to be established for individuals of different age groups.

### Limitations

We only selected the PT values under a single angular velocity, namely 60°/s, for regression analysis because the PT values measured at this velocity were highly accurate and repeatable and is the most commonly used speed for knee joint isokinetic strength test in clinical and research. However, we must admit that this also limits the application of this prediction equation in other angular velocity testing schemes. In addition, our study only included a limited set of easily obtainable predictive factors. Some factors that may affect the PT value, such as regular sports activities, are not considered in the equation due to their quantitative complexity. Furthermore, this study is not applicable to children and the elderly. Considering that there are some difficulties in collecting isokinetic test data for children and the elderly, namely that children often cannot accurately complete the entire testing process, while the elderly often suffer from underlying diseases that are not suitable for isokinetic muscle strength testing, this study did not include these two groups of people. These limitations should be addressed in future research.

## Conclusion

Overall, compared to previous prediction equations, this study provides more accurate prediction equations for the isokinetic strength of knee flexion and extension in young and middle-aged non-athletes, with a relatively lower average bias in the equation. The equations developed in this study provide clinicians with a simple, practical, effective, and more accurate method for predicting patient performance on isokinetic testing equipment before the injury, helping clinicians to more accurately evaluate patient muscle strength, identify early loss of force, and establish more appropriate muscle strengthening goals.

## Data Availability

This trial has been registered in the China clinical trial registration center registration (https://www.chictr.org.cn), and the registration number is ChiCTR2200065772 (Registration date: 15/11/2022). The datasets generated and/or analyzed during the current study are available in the Resman clinical trial management public platform repository (http://www.medresman.org.cn).

## Abbreviations

PT: Peak torque
EPT: Extension peak torque
FPT: Flexion peak torque
ICC: Intraclass correlation coefficients
CI: Confidence interval
